# Dynamic lactate clearance within the first 48 hours predicts pressure injury during ECMO support: a longitudinal cohort study

**DOI:** 10.3389/fmed.2026.1944292

**Published:** 2026-10-14

**Authors:** Zimei Niu, Jing Zhang, Zhenjie Hu, Anqing Li, Lingyan Ma, Songqi Wei

**Affiliations:** Department of Coronary Care Unit, Fuwai Central China Cardiovascular Hospital, Zhengzhou, China

**Keywords:** critical care, extracorporeal membrane oxygenation, lactate clearance, pressure injury, risk prediction, tissue perfusion

## Abstract

**Backgrounds:**

This study aimed to investigate the association between dynamic lactate clearance and the development of pressure injuries in adult patients undergoing extracorporeal membrane oxygenation (ECMO) support and to evaluate its incremental value for early risk prediction.

**Mehods:**

This was a retrospective longitudinal cohort study. A total of 317 adult patients receiving ECMO support were enrolled. Lactate concentrations were measured at ECMO initiation and at 6, 24, and 48 h thereafter. Lactate clearance rates were calculated for each corresponding time point. Multivariable logistic regression analysis was performed to identify independent risk factors for pressure injury development. The predictive performance of lactate clearance was assessed using receiver operating characteristic (ROC) curves, five-fold cross-validation, net reclassification improvement (NRI), and integrated discrimination improvement (IDI).

**Results:**

Pressure injuries occurred in 95 patients (30.0%). Compared with those without pressure injuries, affected patients exhibited significantly lower lactate clearance rates throughout the first 48 h post-ECMO initiation (all *P* < 0.001). Independent predictors of pressure injury included higher body mass index, elevated vasoactive-inotropic score, VA-ECMO mode, and concomitant renal replacement therapy. The baseline clinical model yielded an area under the curve (AUC) of 0.710. Incorporating lactate clearance significantly improved model discrimination, with AUCs increasing to 0.796, 0.825, and 0.899 for the 6-hour, 24-hour, and 48-hour models, respectively. Consistent enhancements in predictive accuracy were confirmed by cross-validation, NRI, and IDI analyses.

**Conclusions:**

Impaired lactate clearance is independently associated with pressure injury development in critically ill adults supported by ECMO. Dynamic monitoring of lactate clearance substantially enhances risk stratification beyond traditional clinical parameters.

## 1 Introduction

For Extracorporeal membrane oxygenation (ECMO) has become an established rescue therapy for patients with severe cardiopulmonary failure (1, 2). As survival continues to improve, increasing attention has shifted toward complications that may adversely affect recovery and long-term outcomes (3, 4).

Pressure injury is one of the most common complications in critically ill patients, affecting approximately 10%–25% of ICU patients (5–7). In ECMO-supported populations, reported incidence rates are considerably higher, generally ranging from 20% to 40% (8). Prolonged immobilization, deep sedation, mechanical ventilation, vasopressor exposure, and hemodynamic instability place ECMO patients at substantial risk of pressure-related tissue injury (8). Beyond local tissue damage, pressure injuries are associated with infection, prolonged hospitalization, increased healthcare costs, and poorer clinical outcomes (9, 10).

Although pressure injuries have traditionally been attributed to sustained external pressure, growing evidence suggests that impaired tissue perfusion is a critical determinant of tissue susceptibility to injury (11–13). This concept may be especially relevant in ECMO-supported patients. While ECMO can restore systemic oxygen delivery and stabilize macrocirculatory parameters, microcirculatory recovery is often delayed, resulting in persistent tissue hypoxia despite apparent hemodynamic improvement (14–17). Such microvascular dysfunction has been increasingly recognized as an important contributor to adverse outcomes in critical illness (14).

Serum lactate is widely used as a marker of tissue hypoperfusion and metabolic stress (18–21). However, a single lactate measurement provides only a static assessment of physiological status. In contrast, lactate clearance reflects the dynamic resolution of oxygen debt and recovery of tissue perfusion over time. Impaired lactate clearance has been associated with worse outcomes in various shock states and among patients receiving ECMO support (20, 22, 23). Existing clinical evidence on dynamic lactate monitoring has predominantly focused on early resuscitation timeframes within the first 6 to 24 h (23, 24). However, in critically ill patients undergoing ECMO support, macro- and microcirculatory recovery is frequently delayed due to severe pre-existing tissue oxygen debt, ischemia–reperfusion responses, and complex shock pathophysiology (25). Consequently, the significance of lactate clearance kinetics beyond 24 h remains unclear. Extending assessment to 48 h after ECMO initiation may better capture persistent microvascular dysfunction, an underrecognized contributor to pressure injury development. The first 24–48 h represent a critical window for hemodynamic stabilization and microcirculatory recovery.

Given that inadequate lactate clearance may reflect persistent microvascular dysfunction and unresolved tissue hypoxia, it may serve as a useful biomarker for identifying ECMO patients at high risk of pressure injury formation. Therefore, this study aimed to investigate the association between dynamic lactate clearance during the first 48 h and pressure injury development in adult patients receiving ECMO support. We further evaluated whether the addition of lactate clearance could improve the predictive performance of conventional pressure injury risk assessment models.

## 2 Methods

### 2.1 Study design and population

This retrospective cohort study was conducted in the intensive care unit of a tertiary academic medical center. Consecutive adult patients who received ECMO support between January 2018 and December 2025 were screened for eligibility.

Patients were included if they met all of the following criteria: (1) age ≥18 years; (2) receipt of venoarterial (VA) ECMO or venovenous (VV) ECMO support; (3) availability of serial serum lactate measurements at ECMO initiation and during the first 48 h of ECMO support; and (4) complete pressure injury surveillance records during hospitalization.

Patients were excluded if they: (1) had a documented pressure injury before ECMO initiation; (2) died within 48 h after ECMO initiation; (3) received ECMO support for less than 48 h; (4) had missing key clinical variables required for multivariable analyses; or (5) had pre-existing skin defects related to burns, trauma, or surgical wounds that could interfere with pressure injury assessment; (6) had pressure injury within 48 h after ECMO initiation.

The study protocol was approved by the Institutional Review Board of our Hospital and was conducted in accordance with the principles of the Declaration of Helsinki. Owing to the retrospective design and the use of anonymized data, the requirement for informed consent was waived by the Board.

### 2.2 Standardized pressure injury prevention care

All enrolled patients received standardized pressure injury prevention care in accordance with institutional ICU protocols, which included the routine use of dynamic pressure-relieving air mattresses, skin barrier protectants, and planned body repositioning every 2 h when clinically feasible.

### 2.3 Data collection

Baseline demographic variables included age, sex, and body mass index (BMI). Variables reflecting illness severity, nutritional status, and circulatory function at ECMO initiation were collected, including Sequential Organ Failure Assessment (SOFA) score, vasoactive-inotropic score (VIS), serum albumin concentration, baseline serum lactate concentration, ECMO mode (VA-ECMO or VV-ECMO), the requirement for renal replacement therapy (RRT), ECMO-related variables included indication for ECMO, support mode, ECMO duration and baseline ECMO flow. The implementation of physical therapy (PT) and occupational therapy (OT) interventions was recorded. Baseline mean arterial pressure (MAP) was recorded. Laboratory measurements were obtained according to routine institutional practice. Serum lactate concentrations were recorded at ECMO initiation (T0) and at approximately 6 h (T6), 24 h (T24), and 48 h (T48) after cannulation.

### 2.4 Definition of dynamic lactate clearance

The primary exposure variable was dynamic lactate clearance during the first 48 h of ECMO support. Lactate clearance at each time point was calculated using the following formula: Lactate clearance (%) = [(Lactate at T0−Lactate at Tx)/Lactate at T0] × 100, where Tx represented the lactate concentration measured at 6, 24, or 48 h after ECMO initiation. Accordingly, three dynamic lactate clearance variables were generated: 6-hour lactate clearance (LC-6 h), 24-hour lactate clearance (LC-24 h), 48-hour lactate clearance (LC-48 h).

Positive values indicated a reduction in serum lactate concentration compared with baseline, reflecting recovery of tissue perfusion and metabolic homeostasis. Lower lactate clearance values indicated persistent tissue hypoperfusion and impaired metabolic recovery.

### 2.5 Outcomes

The primary outcome was the development of a pressure injury during ECMO support. Pressure injuries was identified and staged by certified wound care nurses according to the National Pressure injuries Advisory Panel (NPIAP) classification system. All stages of pressure injuries were included in the analysis. For patients who developed multiple pressure injuries, only the first documented pressure injury event was considered. Patients were subsequently classified into a pressure injury group and a non-pressure injury group according to whether a pressure injury occurred during ECMO support.

### 2.6 Statistical analysis

Statistical analyses were performed using R software (version 4.4.3). Continuous variables were tested for normality using the Shapiro–Wilk test and are presented as mean ± standard deviation (SD) or median [interquartile range (IQR)], as appropriate. Comparisons between groups were performed using Student's t test or the Mann–Whitney U test for continuous variables and the *χ*^2^ test or Fisher's exact test for categorical variables. Patients were stratified according to pressure injury occurrence during ECMO support. Variables with a *P* value <0.10 in univariable analyses, together with clinically relevant covariates, were entered into multivariable logistic regression models to identify independent predictors of pressure injury development. Results are reported as odds ratios (ORs) with 95% confidence intervals (CIs).

A baseline clinical prediction model was established using only the statistically significant independent clinical risk factors identified in the multivariable analysis. Baseline lactate concentration and dynamic lactate clearance variables (6-hour, 24-hour, and 48-hour lactate clearance) were then added separately to evaluate their incremental predictive value. Model discrimination was assessed using receiver operating characteristic (ROC) curve analysis and the area under the ROC curve (AUC). Internal validation was performed using five-fold stratified cross-validation, with cross-validated AUC (CV-AUC) reported. Improvements in predictive performance were further quantified using changes in AUC (*Δ*AUC), net reclassification improvement (NRI), and integrated discrimination improvement (IDI).

All statistical tests were two-sided, and a *P* value <0.05 was considered statistically significant.

## 3 Results

### 3.1 Patient characteristics

A total of 834 adult patients who received ECMO support were retrospectively screened for eligibility. After applying the inclusion and exclusion criteria, 317 patients were included in the final analysis. The reasons for exclusion and corresponding numbers were as follows: 86 patients were excluded due to documented pressure injuries before ECMO initiation; 72 patients died within 48 h after ECMO initiation; 68 patients received ECMO support for less than 48 h; 154 patients had missing key clinical variables required for multivariable analyses; 125 patients had pre-existing skin defects related to burns, trauma, or surgical wounds that could interfere with pressure injury assessment; and 12 patients developed pressure injuries within 48 h after ECMO initiation. During ECMO treatment, 95 patients developed pressure injuries, yielding an overall incidence of 30.0%. Baseline characteristics stratified by pressure injury occurrence are presented in Table 1. Compared with patients who remained free of pressure injuries, those who subsequently developed pressure injuries had a significantly higher body mass index (26.3 ± 4.5 vs. 25.0 ± 4.8 kg/m^2^, *P* = 0.019), higher vasoactive-inotropic scores (23.5 ± 10.9 vs. 18.5 ± 8.9, *P* < 0.001), and were more likely to require renal replacement therapy (50.5% vs. 34.7%, *P* = 0.012). The proportion of patients supported with VA-ECMO was also significantly greater in the pressure injury group (*P* = 0.001). Patients who developed pressure injuries had higher baseline SOFA scores than those without pressure injuries (8.5 ± 2.8 vs. 7.8 ± 2.7, *P* = 0.040). No significant differences were observed in age, sex, serum albumin concentration, ECMO duration, baseline MAP, baseline ECMO flow, mechanical ventilation use or intervention of PT and OT between the two groups.

**Table 1:** Baseline characteristics of ECMO patients stratified by pressure injury occurrence.

Variable	No Pressure injury (*n* = 222)	Pressure injury (*n* = 95)	*P*-value
Age, years	51.8 ± 14.1	53.5 ± 14.5	0.485
Gender, male, *n* (%)	120 (54.1)	45 (47.4)	0.333
BMI, kg/m^2^	25.0 ± 4.8	26.3 ± 4.5	0.019
VIS score	18.5 ± 8.9	23.5 ± 10.9	<0.001
ECMO duration, hours	148.9 ± 70.3	153.2 ± 82.0	0.877
Baseline ECMO flow, L/min	3.8 ± 0.6	3.9 ± 0.8	0.491
Mean Arterial Pressure, mmHg	68.2 ± 9.1	67.5 ± 8.9	0.521
Albumin, g/L	32.5 ± 6.1	32.7 ± 5.8	0.623
SOFA score	7.8 ± 2.7	8.5 ± 2.8	0.040
VA-ECMO mode, *n* (%)	100 (45%)	62 (65.3%)	0.001
RRT, *n* (%)	77 (34.7)	48 (50.5)	0.012
Mechanical Ventilation, *n* (%)	202 (91.0)	89 (93.7)	0.423
Physical/Occupational therapy, *n* (%)	29 (13.1)	14 (14.7)	0.690

BMI, body mass index; VIS, vasoactive-inotropic score; ECMO, extracorporeal membrane oxygenation; SOFA, sequential organ failure assessment; RRT, renal replacement therapy; VA, venoarterial; VV, venovenous.

### 3.2 Characteristics of pressure injuries

Clinical characteristics of pressure injuries are summarized in Table 2. Most pressure injuries developed during the first week of ECMO support, with 61.1% occurring between days 4 and 7 and a median onset time of 5.5 days (IQR, 4.0–7.5 days). The sacral/coccygeal region was the most frequently affected site, accounting for 50.5% of all ulcers, followed by the heels (22.1%) and cannulation- or device-related sites (14.7%). Among the 95 patients (30.0%) who developed pressure injuries, most were early-stage or deep tissue injuries according to NPIAP staging. Stage 1 was most common [33 (34.7%)], followed by deep tissue injuries [22 (23.2%)] and Stage 2 [19 (20.0%)]. Stage 3 and 4 injuries were uncommon [11 (11.6%) and 3 (3.2%), respectively], as were unstageable injuries [7 (7.4%)].

**Table 2:** Clinical characteristics of ECMO-related pressure injuries.

Characteristics	Patients with PI (*n* = 95)	Percentage (%)
**Total cohort (*N*** **=** **317)**	95	30.0%
Time to PI Development		
≤ 3 days	14	14.7%
4–7 days	58	61.1%
> 7 days	23	24.2%
Median time (IQR), days	5.5 (4.0, 7.5)	—
**Anatomical Location**		
Sacrum/Coccyx	48	50.5%
Heels	21	22.1%
Cannulation/Device sites	14	14.7%
Occiput	8	8.4%
Others (Trochanter, Scapula)	4	4.2%
**NPIAP Staging**		
Stage 1	33	34.7%
Stage 2	19	20.0%
Stage 3	11	11.6%
Stage 4	3	3.2%
Unstageable	7	7.4%
Deep Tissue Pressure injuries	22	23.2%

PI, pressure injury.

### 3.3 Independent clinical risk factors for pressure injury development

Multivariable logistic regression analysis identified several independent predictors of pressure injury occurrence (Table 3). Higher BMI was associated with an increased risk of pressure injury formation (OR, 1.063; 95% CI, 1.009–1.120; *P* = 0.021). Similarly, each incremental increase in VIS was independently associated with greater pressure injury risk (OR, 1.039; 95% CI, 1.010–1.069; *P* = 0.007).

**Table 3:** Multivariable logistic regression analysis of baseline clinical risk factors for pressure injury.

Variates	OR	95% CI	*P*-value
BMI	1.063	1.009–1.120	0.021
VIS score	1.039	1.010–1.069	0.007
VA-ECMO mode	2.202	1.319–3.674	0.002
RRT	1.939	1.166–3.225	0.011

BMI, body mass index; VIS, vasoactive-inotropic score; VA-ECMO; venoarterial extracorporeal membrane oxygenation; RRT, renal replacement therapy.

Patients receiving VA-ECMO had more than twice the odds of developing pressure injuries compared with those supported with VV-ECMO (OR, 2.202; 95% CI, 1.319–3.674; *P* = 0.002). Requirement for renal replacement therapy was also independently associated with pressure injury development (OR, 1.939; 95% CI, 1.166–3.225; *P* = 0.011).

### 3.4 Dynamic lactate profiles and lactate clearance

Longitudinal lactate measurements demonstrated distinct metabolic trajectories between groups (Table 4, Figure 1). At ECMO initiation, patients who later developed pressure injuries already exhibited higher serum lactate concentrations than those who did not (4.05 ± 1.80 vs. 3.50 ± 1.50 mmol/L, *P* = 0.029). Following ECMO initiation, lactate levels progressively decreased in the non-pressure injury group, whereas persistently elevated lactate concentrations were observed among patients who subsequently developed pressure injuries. These between-group differences became more pronounced over time and remained highly significant at 6, 24, and 48 h (all *P* < 0.001).

**Table 4:** Longitudinal lactate profiles and clearance rates of ECMO patients.

Parameter	No Pressure injury Group (*n* = 222)	Pressure injury Group (*n* = 95)	*P*-value
Serum Lactate (mmol/L)			
T0 (Baseline)	3.5 ± 1.5	4.05 ± 1.8	0.029
6h	3.02 ± 1.31	3.82 ± 1.70	<0.001
24h	2.78 ± 1.27	3.73 ± 1.71	<0.001
48h	2.41 ± 1.19	3.77 ± 1.74	<0.001
Lactate Clearance (%)			
6h	14.7 ± 11.4	5.7 ± 9.5	<0.001
24h	21.5 ± 15.4	7.8 ± 13.0	<0.001
48h	31.8 ± 18.4	6.8 ± 14.0	<0.001

**Figure 1:**
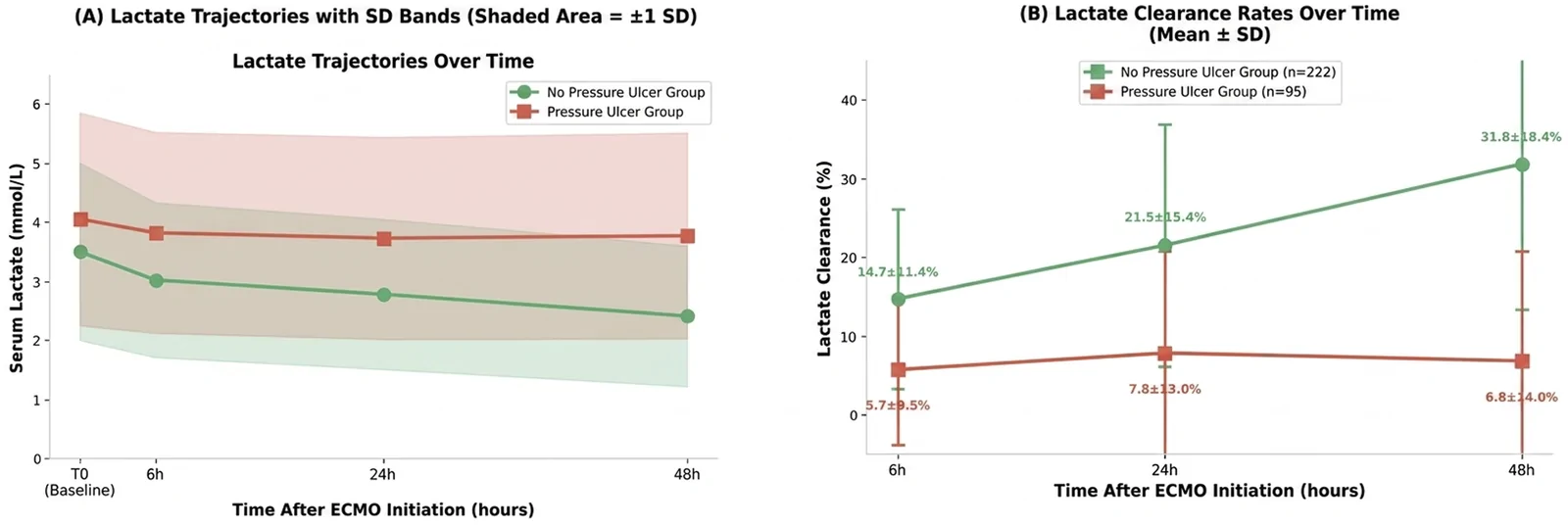
Dynamic changes in serum lactate concentration and lactate clearance. **(A)** Mean serum lactate concentrations from ECMO initiation (T0) to 48 h in patients with and without pressure ulcers. Shaded areas represent ± SD. **(B)** Lactate clearance at 6, 24, and 48 h after ECMO initiation. Data are presented as mean ± SD. ECMO, extracorporeal membrane oxygenation; SD, standard deviation; T0, ECMO initiation.

Consistent with these findings, lactate clearance was substantially impaired in patients who developed pressure injuries. At 6 h, lactate clearance was nearly threefold lower in the pressure injury group (5.7% ± 9.5% vs. 14.7% ± 11.4%, *P* < 0.001). The magnitude of divergence increased further at 24 h (7.8% ± 13.0% vs. 21.5% ± 15.4%, *P* < 0.001) and reached its greatest extent at 48 h (6.8% ± 14.0% vs. 31.8% ± 18.4%, *P* < 0.001). These findings suggest that persistent impairment of lactate clearance, rather than a single elevated lactate measurement, was strongly associated with subsequent pressure injury development.

### 3.5 Incremental predictive value of dynamic lactate clearance

The baseline clinical model incorporating BMI, VIS, ECMO mode, and RRT demonstrated moderate discrimination for pressure injury prediction (AUC, 0.710; CV-AUC, 0.691 ± 0.040) (Table 5, Figure 2). Addition of baseline lactate modestly improved model performance, increasing the AUC to 0.743 (*Δ*AUC = 0.033). In contrast, incorporation of lactate clearance variables produced progressively greater improvements in discrimination. The AUC increased to 0.796 with 6-hour lactate clearance, 0.825 with 24-hour lactate clearance, and 0.899 with 48-hour lactate clearance. Similar improvements were observed in internally validated performance, with CV-AUC values rising from 0.691 to 0.788, 0.806, and 0.897, respectively.

**Table 5:** Incremental predictive value of dynamic lactate clearance.

Model	AUC	*Δ*AUC	CV-AUC (5-fold)	NRI	IDI	OR	*P*-value
Baseline Model	0.710	—	0.691 ± 0.040	—	—	—	—
+ T0 Lactate	0.743	0.033	0.723 ± 0.043	0.389	0.034	1.344	0.002
+ 6 h Clearance	0.796	0.086	0.788 ± 0.048	0.651	0.098	0.926	<0.001
+ 24 h Clearance	0.825	0.115	0.806 ± 0.039	0.768	0.155	0.935	<0.001
+ 48 h Clearance	0.899	0.189	0.897 ± 0.041	1.246	0.320	0.911	<0.001

AUC, area under the receiver operating characteristic curve; *Δ*AUC, absolute difference in AUC compared with the baseline model; CV-AUC, cross-validated AUC using 5-fold stratified cross-validation (mean ± standard deviation); NRI, net reclassification improvement; IDI, integrated discrimination improvement; OR, odds ratio from multivariable logistic regression analysis.

**Figure 2:**
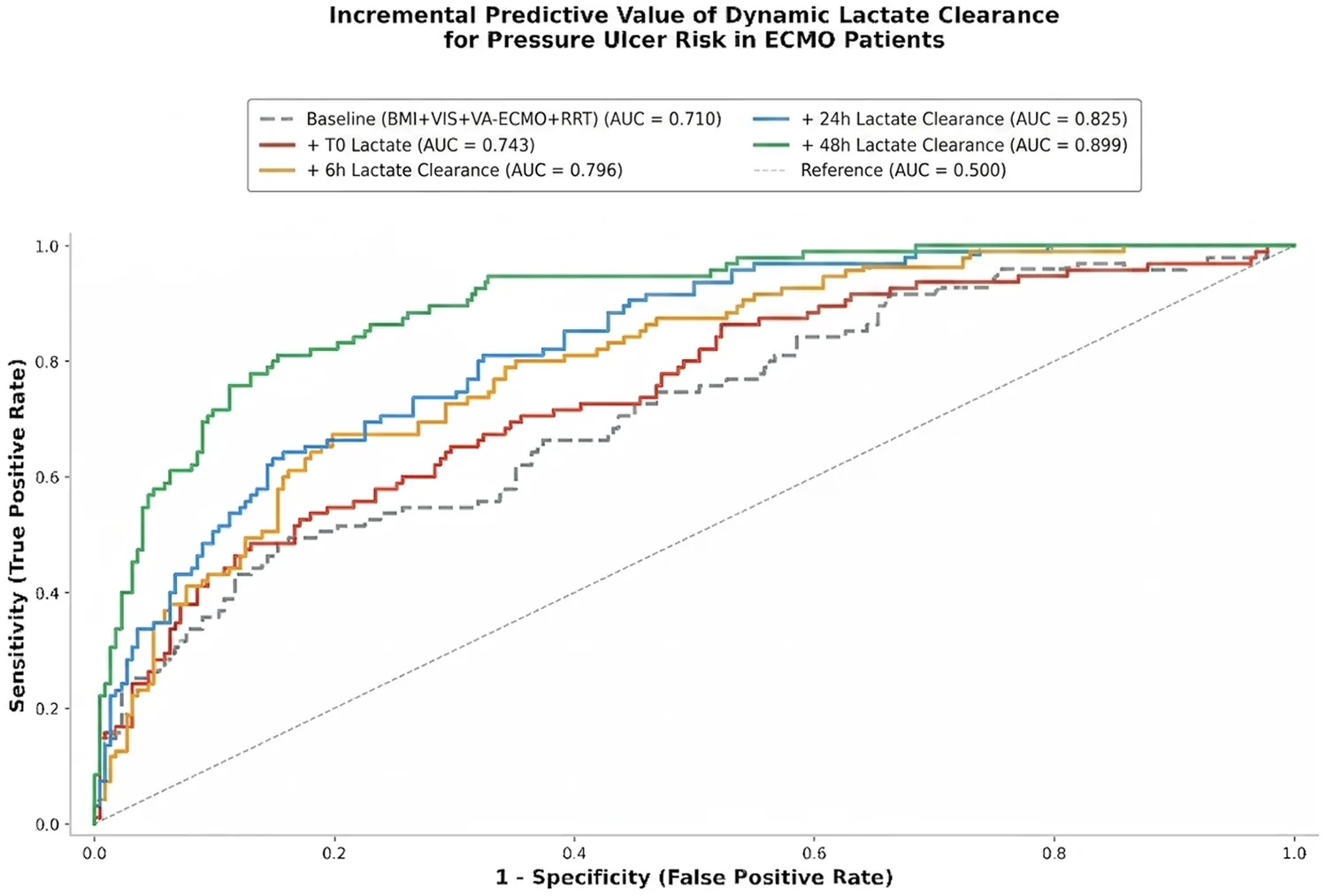
Receiver operating characteristic (ROC) curves for predicting pressure ulcer development. The dashed gray line represents the baseline clinical model (BMI, VIS, VA-ECMO, and RRT). Solid lines represent model performance after adding baseline lactate (T0) or lactate clearance at 6, 24, and 48 h. Discrimination was assessed using the area under the curve (AUC).

Risk reclassification analyses further supported the incremental value of dynamic lactate clearance. Compared with the baseline model, the net reclassification improvement increased from 0.651 for 6-hour clearance to 1.246 for 48-hour clearance, while the integrated discrimination improvement rose from 0.098 to 0.320. In multivariable analyses, higher lactate clearance at each time point was independently associated with a lower risk of pressure injury development (all *P* < 0.001). Overall, the predictive contribution of lactate clearance strengthened progressively from 6 to 48 h, with 48-hour lactate clearance providing the greatest improvement in model discrimination and risk stratification.

## 4 Discussion

In this retrospective cohort study of adult patients receiving ECMO support, we found that impaired lactate clearance during the first 48 h after ECMO initiation was strongly associated with subsequent pressure injury development. Patients who developed pressure injuries exhibited persistently elevated lactate concentrations and markedly reduced lactate clearance throughout the early phase of ECMO support. Importantly, the predictive value of lactate clearance increased progressively over time, with 48-hour lactate clearance demonstrating the strongest association with pressure injury occurrence and providing substantial incremental predictive value beyond conventional clinical risk factors. Incorporation of dynamic lactate clearance improved model discrimination from an AUC of 0.710 to 0.899 and resulted in significant gains in risk reclassification and internal validation performance.

These findings extend the current understanding of pressure injury pathogenesis in critically ill patients receiving ECMO. Traditionally, pressure injuries have been viewed primarily as a consequence of prolonged pressure exposure, immobility, and mechanical tissue deformation (26, 27). Although these factors remain important, our results support a broader conceptual framework in which systemic perfusion failure and persistent microcirculatory dysfunction play a central role in determining tissue vulnerability (14, 28). Notably, most pressure injuries occurred within the first week of ECMO support, a period characterized by profound physiological instability and ongoing recovery from circulatory shock. The observation that impaired lactate clearance preceded pressure injury development suggests that inadequate restoration of tissue perfusion may contribute to tissue breakdown well before clinically apparent skin injury becomes evident (23, 29, 30).

A particularly noteworthy finding was the progressive increase in predictive performance from 6-hour to 48-hour lactate clearance. While baseline lactate and early lactate measurements were associated with pressure injury risk, their predictive contribution was substantially lower than that of later clearance metrics. This temporal pattern likely reflects the evolving pathophysiology of shock recovery. Immediately after ECMO initiation, lactate kinetics may be influenced by multiple factors, including pre-existing oxygen debt, ischemia–reperfusion injury, catecholamine exposure, hepatic dysfunction, and altered metabolic clearance. Consequently, early lactate values may incompletely reflect actual tissue reperfusion. In contrast, persistent impairment of lactate clearance at 24 and 48 h is more likely to indicate ongoing microvascular dysfunction and unresolved tissue hypoxia. The superior performance of 48-hour lactate clearance therefore suggests that sustained hypoperfusion, rather than the severity of the initial insult alone, is a key determinant of pressure injury development during ECMO support.

The biological plausibility of these findings is supported by growing evidence regarding the dissociation between macrocirculatory stabilization and microcirculatory recovery in critical illness (31). ECMO effectively restores systemic oxygen delivery and improves global hemodynamic parameters; however, normalization of microvascular blood flow is frequently delayed (14, 15). Experimental and clinical studies have demonstrated persistent abnormalities in capillary perfusion, endothelial function, and tissue oxygen extraction despite apparently adequate systemic circulation (15, 32). Under such conditions, pressure-loaded tissues may remain vulnerable to ischemic injury even when conventional hemodynamic targets have been achieved. Lactate clearance may therefore serve as a readily available surrogate marker of this hidden perfusion deficit, capturing the cumulative effects of impaired oxygen delivery, endothelial dysfunction, and microvascular failure that are not reflected by traditional vital signs.

Our findings are further supported by the independent associations observed for VIS, VA-ECMO support, and renal replacement therapy. Higher vasoactive-inotropic requirements likely reflect greater circulatory instability and vasoconstrictor exposure, both of which may compromise skin and soft tissue perfusion (33). Similarly, patients requiring VA-ECMO represent a population with more severe cardiogenic shock and profound systemic hypoperfusion (14). Renal replacement therapy may additionally identify patients with greater illness severity and multiorgan dysfunction (34) Collectively, these factors support the hypothesis that pressure injury formation during ECMO is closely linked to the severity and duration of systemic perfusion abnormalities.

From a clinical perspective, these findings have important implications for risk stratification and preventive care. Current pressure injury assessment tools primarily focus on demographic characteristics, mobility status, nutritional factors, and nursing-related variables (35). Although valuable, such approaches may inadequately capture the dynamic physiological disturbances that characterize ECMO patients. Our results suggest that incorporation of lactate clearance into pressure injury risk assessment could substantially improve early identification of high-risk individuals. While 48-hour lactate clearance achieved the highest predictive accuracy, the strong performance observed at 24 h may be particularly relevant clinically, as it provides an actionable time window during which intensified preventive interventions can still be implemented. Patients demonstrating poor lactate clearance could potentially benefit from enhanced pressure redistribution strategies, more frequent repositioning, intensified skin surveillance, and optimization of tissue perfusion targets.

Nevertheless, the biological interpretation of lactate clearance as a marker of microvascular hypoperfusion warrants cautious consideration. Hyperlactatemia may arise from multiple mechanisms beyond impaired tissue perfusion, including reduced hepatic lactate metabolism, stress-induced glycolysis, and drug-related metabolic alterations. To mitigate the potential impact of these confounding factors, we incorporated several adjustment strategies in our analysis. First, multivariable regression models were adjusted for overall organ dysfunction using the SOFA score, which includes total bilirubin as an indicator of hepatic function, as well as renal replacement therapy and vasoactive support intensity assessed by the VIS score. Second, by evaluating dynamic lactate clearance (percentage change over 48 h) rather than isolated lactate concentrations at a single time point, we minimized the influence of baseline metabolic heterogeneity unrelated to tissue hypoperfusion. Nevertheless, given the retrospective design of this study, we could not completely exclude all potential non-hypoperfusion-related determinants of lactate kinetics, and residual unmeasured confounding may still have affected our findings.

There are several limitations in this study. First, this was a retrospective single-center study, and residual confounding cannot be completely excluded despite multivariable adjustment. In particular, detailed information on rehabilitation protocols, cannulation sites, longitudinal trajectories of ECMO flow adjustments, and continuous monitoring data for MAP or other advanced hemodynamic parameters could not be fully captured or quantified, which may have influenced the observed outcomes. Second, the observational design precludes causal inference between impaired lactate clearance and pressure injury development. Third, lactate clearance was used as an indirect surrogate of tissue perfusion, whereas direct assessments of microcirculatory function were unavailable. Fourth, pressure injury prevention practices and nursing interventions may have varied among patients and could not be fully captured in the analysis. Finally, competing-risk bias may have influenced our findings. Patients receiving ECMO are at substantial risk of early mortality, withdrawal of care, or discontinuation of ECMO support, all of which may preclude subsequent pressure injury development. Because pressure injury occurrence was analyzed as a binary outcome and competing events were not explicitly modeled, the incidence of pressure injuries may have been underestimated in the sickest patients. Future studies incorporating time-to-event and competing-risk analyses are warranted to validate our findings. Despite these limitations, the study provides novel evidence linking impaired lactate clearance to pressure injury development during ECMO support and highlights the potential value of dynamic perfusion monitoring for early risk stratification.

In conclusion, impaired lactate clearance was independently associated with pressure injury development among adult patients receiving ECMO support. The predictive value of lactate clearance increased progressively from 6 to 48 h after ECMO initiation, suggesting that persistent microcirculatory dysfunction and unresolved tissue hypoperfusion play a critical role in pressure injury pathogenesis. Dynamic lactate clearance may represent a simple and widely available biomarker for early risk stratification and targeted prevention of pressure injuries in this high-risk population.

## Data Availability

Publicly available datasets were analyzed in this study. This data can be found here: The datasets are available from the corresponding author on reasonable request.
